# Cardiorespiratory Interaction and Autonomic Sleep Quality Improve during Sleep in Beds Made from *Pinus cembra* (Stone Pine) Solid Wood

**DOI:** 10.3390/ijerph18189749

**Published:** 2021-09-16

**Authors:** Vincent Grote, Matthias Frühwirth, Helmut K. Lackner, Nandu Goswami, Markus Köstenberger, Rudolf Likar, Maximilian Moser

**Affiliations:** 1 Physiology Division, Otto Loewi Research Center for Vascular Biology, Immunology and Inflammation, Medical University of Graz, 8010 Graz, Austria; helmut.lackner@medunigraz.at (H.K.L.); nandu.goswami@medunigraz.at (N.G.); max.moser@medunigraz.at (M.M.); 2 Human Research Institute of Health Technology and Prevention Research, 8160 Weiz, Austria; matthias.fruehwirth@humanresearch.at; 3 Ludwig Boltzmann Institute for Rehabilitation Research, Ludwig Boltzmann Gesellschaft, 1140 Vienna, Austria; 4 Department of Anesthesiology and Intensive Care Medicine, Klinikum Klagenfurt am Woerthersee, 9020 Klagenfurt, Austria; markus.koestenberger@kabeg.at (M.K.); Rudolf.Likar@kabeg.at (R.L.); 5 Chair for Palliative Medicine, Faculty of Medicine, Sigmund Freud University, 1020 Vienna, Austria

**Keywords:** *Pinus cembra*, wood, sleep, autonomic nervous system, vagal activity, healing environment

## Abstract

Cardiorespiratory interactions (CRIs) reflect the mutual tuning of two important organismic oscillators—the heartbeat and respiration. These interactions can be used as a powerful tool to characterize the self-organizational and recreational quality of sleep. In this randomized, blinded and cross-over design study, we investigated CRIs in 15 subjects over a total of 253 nights who slept in beds made from different materials. One type of bed, used as control, was made of melamine faced chipboard with a wood-like appearance, while the other type was made of solid wood from stone pine (*Pinus cembra*). We observed a significant increase of vagal activity (measured by respiratory sinus arrhythmia), a decrease in the heart rate (as an indicator of energy consumption during sleep) and an improvement in CRIs, especially during the first hours of sleep in the stone pine beds as compared to the chipboard beds. Subjective assessments of study participants’ well-being in the morning and sub-scalar assessments of their intrapsychic stability were significantly better after they slept in the stone pine bed than after they slept in the chipboard bed. Our observations suggest that CRIs are sensitive to detectable differences in indoor settings that are relevant to human health. Our results are in agreement with those of other studies that have reported that exposure to volatile phytochemical ingredients of stone pine (α-pinene, limonene, bornyl acetate) lead to an improvement in vagal activity and studies that show a reduction in stress parameters upon contact with solid wood surfaces.

## 1. Introduction

Humans perceive wood as being practical, aesthetic and climate friendly. Psychologically, wood is also considered to be warmer, more comforting, relaxing and inviting as compared to wood laminate by both expert and non-expert subjects [1,2]. In contrast to earlier views, newer studies have shown that wood surfaces suppress microbial activity better than plastic surfaces [3] due to their inherent antiviral, antifungal, antibacterial [4,5] and antistatic qualities [6]. Therefore, wood can even be used safely in a hospital environment [4,7,8,9].

A growing body of scientific evidence points to the potential health effects of natural environments that have the potential to modulate, for example, immunological responses, such as those caused by biogenic volatile organic compounds (BVOCs) [10,11]. The positive effects of contact with nature and green spaces range from indirect effects (e.g., increased physical activity) to direct psychological and physiological effects and longer life expectancies.

Stone pine (*Pinus cembra*) is a needle-leaved tree in the pine family (Pinaceae) with yellow–red heartwood and a characteristic fragrant smell. Traditionally, its wood has been used in certain regions of Austria (Tyrol), North Italy, South Germany and Switzerland as a preferred material for making beds, cradles and interior wall paneling in in living rooms. Volatile components of conifer resins, such as α-pinene and limonene, have been reported to stimulate vagal activity [12]. Touching conifer wood (e.g., the Hinoki cypress (*Chamaecyparis obtusa*)) with the palms [13] or the soles of the feet [14] results in similar effects. Furthermore, relaxing effects as well as reductions in an aroused state have been seen using another component of pine resin, bornyl acetate [15]. For instance, 80% of the subjects in a study showed increased vagal activity in a forest surrounding as compared to in an urban surrounding with similar physical characteristics [16].

Cardiorespiratory interactions (CRIs) result from the optimization and cooperation of two important organismic oscillators: cardiac and respiratory cycles. Whereas the heartbeat is generated autonomously via the atrial sinus node, its rate is modulated by autonomous influences from circulatory centers in the brain. In contrast, the onset of inspiration and expiration is controlled directly by gating from respiratory centers in the brainstem [17] and is weakly coupled to the cardiac cycle [18]. Both oscillators are connected via brainstem neurons and interact mainly during periods of rest, and especially during non-REM sleep. During work and daily activity, the cooperation between the oscillators is reduced and less coordination is seen [19]. Similar reductions in numbers of interactions are also seen in depressed subjects, even at rest [20]. Several studies show why mutually tuning the heartbeat and respiration is energy-efficient and evolutionarily beneficial [21]. This cooperation between heartbeat and respiration can be observed and quantified, e.g., by examining the amount of phase coupling or coherence [22], the respiratory modulation of the heartbeat intervals (respiratory sinus arrhythmia) [23], or the quotient of the heart rate and respiratory rate (pulse–respiration quotient, Qpr) [24].

In this study, we address different aspects of CRIs to investigate the possible influence of different bed materials, such as chipboard or coniferous solid wood (stone pine (*Pinus cembra*)), on the quality of sleep. As mentioned above, sleep is the preferred state during which CRIs occur. Although they are not yet used in traditional sleep staging, CRIs could be used to assess sleep quality in the future. Several studies suggest that a high level of vagal activity, which is visible as a strong respiratory sinus arrhythmia, is an indicator of a highly recreational sleep [25]. Vagal activity also has been found to influence inflammation and to control inflammatory processes in humans [26], which, in turn, can support good sleep [27,28]. Moreover, a strong respiratory sinus arrhythmia is linked with increased post-myocardial infarction survival [29] and with reduced cardiac mortality and even all-cause mortality [23].

In this randomized, blinded and cross-over design study, we investigated the possibility to use CRIs as a measure to describe the quality of sleep. In addition, we investigated whether the bed material promotes a different quality of sleep as estimated by assessments of psychometric and autonomic parameters (i.e., heart rate variability).

## 2. Methods

### 2.1. Subjects

**Figure 1:**
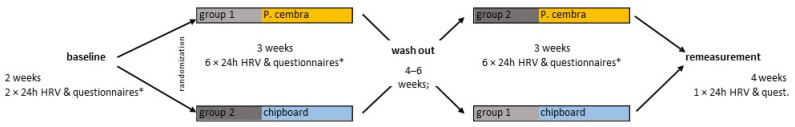
Measure protocol and study design. *. Scales (questionnaires; quest.): PSQI [30], SRS_HRI_ [32], BBS [31] and RSTQ-Basic-24 [33,34].

Fifteen healthy subjects (8 female), aged 17–45 years, from a small town (Weiz) in southeast Austria (12,000 inhabitants) were recruited for the study via public notices and an information session. The average body mass index (BMI) was 22.4 ± 2.5 (19 to 27) kg/m^2^. All subjects were free of medication and reported to be physically fit. The study participants were verbally questioned about subjective somatic complaints at baseline (exclusion criterion: chronic diseases or taking medication) and confirmed by the results of the questionnaires used [30,31,32,33] and the 24 h ECG measurements at baseline (Figure 1).

The subjects used their own mattresses and bedding material (cotton) on all nights of the study under all experimental conditions.

### 2.2. Ethics

The study uses data from healthy subjects for purposes that do not include medical diagnoses, interventions, or treatment. According to Austrian law, an approval from an ethics committee is not mandatory in this context. For the retrospective analysis of the study data, the Carinthian Ethics Commission issued a positive vote on 8 July 2020 (EC number S2020-26). Subjects had given their informed written consent to participate in the study before the first measurements were made and received feedback on their results after completing the study. For participants under the age of 18, the informed consent of a parent and/or legal guardian was obtained. The study protocol adhered to the guidelines of “good clinical practice” (ICH-GCP), followed the Declaration of Helsinki and complied with the regulations of the National Data Protection Act (Section 14 Abs. 1, DSG 2000). The subjects received a financial compensation (€200) for their participation.

### 2.3. Experimental Design

**Figure 2:**
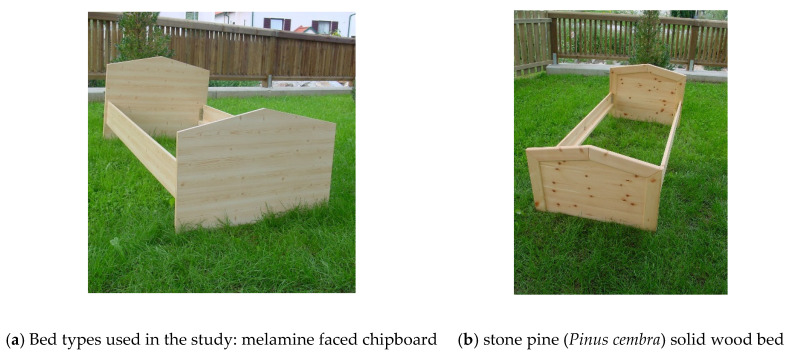
Bed types used in the study: melamine faced chipboard ((**a**), control) and stone pine (*Pinus cembra*) solid wood bed (**b**). Six beds were made of each type. The personal beds of the 15 subjects are not shown.

In a cross-over randomized design study (Figure 1), after undergoing two weeks of measurements in their own beds, the study subjects’ beds were exchanged in their bedrooms either for a melamine-faced chipboard bed (control condition, Figure 2a) or for a wood bed made from solid stone pine wood (Figure 2b). All other bed components such as the mattress and bedding remained unchanged. After three more weeks, during which measurements were performed twice a week, the subjects’ own beds were brought back for 4–6 weeks (washout period). The beds were again alternated to the other condition (stone pine or control bed) for three more weeks, with six measurements performed in total. A final follow-up measurement was performed after the subject slept in their own bed again.

To achieve the best blinding and to avoid a placebo effect, the subjects were informed before the study that the research question would be asked to differentiate between sleep in their own bed and sleep in a new bed. However, no mention of the materials used was made. After completing the study, the real intention of the study was revealed to the participants.

The number of subjects necessary (15) was selected by calculating the expected medium statistical effect size. To increase the accuracy of the measurements, each subject was measured six times over three weeks before, during and after sleeping in each bed.

Measurements were performed twice a week during the weeks of the study, during which the subjects slept either in the chipboard or the solid wood bed, with measurements taken at the same time of day and on the same days of the week to avoid the confounding “Monday” or “Friday” effects that have been demonstrated in other studies [35]. Under each study condition, six 24-h measurements of heart rate variability were performed with the 15 subjects, resulting in a dataset of measurements over 88 nights for each type of bed (two nights were lost under each condition due to a technical failure).

### 2.4. Material

Both bed types were made by the same experienced carpenter (Erich Binder, Weiz, Austria). Material used to make the 12 beds for the study was either low-emission class E1 melamine faced chipboard (MFC, the most widespread material used for furniture sold in Austria, with a global market of 33 billion USD/year) as a control condition or solid stone pine wood, which is traditionally used in some regions of Austria (e.g., Tyrol) and Switzerland. The melamine surface of the control beds was printed with a pattern to make it look like the solid wood beds in terms of its color and surface structure (Figure 2).

### 2.5. Physiological Variables

Physiological parameters were measured over a 24-h period (HR and HRV parameters) [23,36,37,38], during the time scheduled for measurements, and the psychometric parameters were measured in the evening and morning (see Figure 1 and ‘Statistical Evaluation’).

The beat-to-beat heart rate was measured precisely (4000 samples/s, 16-bit) with a miniature Holter-ECG device (ChronoCord, Human Research Institute, Weiz, Austria) for a 24-h period from noon to noon, yielding approx. 106,000 heartbeats for each measurement. Each heart rate time series was inspected by experienced scientists to identify missing data and ectopic beats, which were then removed before further data processing. From the RR time series, heart rate variability measures such as low frequency (LF), high frequency (HF) and very low frequency (VLF) variability measures were computed according to the Task Force recommendations [36]. The autonomic quotient (LF/HF) was computed from LF and HF results. LogRSA, a robust, time domain measure which is especially suitable for the estimation of the respiratory component of HRV and, hence, vagal activity [20,23,39,40,41], was used to calculate vagal activity every five minutes at two-minute increments.

By analyzing the beat-to-beat changes of the RR interval, it is possible to obtain a clinically reliable respiration frequency from the respiratory sinus arrhythmia (RSA) during resting periods. Additionally, as a consequence of respiration-induced diaphragm movements, changes in the electrical axis of the heart can be used to derive the respiration frequency from the ECG [42,43,44]. The pulse–respiration quotient (Qpr) [24] was computed from the respiratory and heartbeat frequencies as described in [42] and [18]. It represents the number of heartbeats in each respiratory cycle.

### 2.6. Psychometric Variables

For each 24 h measurement, the subjects had to keep an activity log and to fill out questionnaires at (pre-) defined times: Before they went to bed, they had to evaluate their stress levels for the previous three days with the RESTQ instrument [33,34] (Pearson, Frankfurt, Germany). In the morning, immediately after getting up, the subjects had to fill in a sleep questionnaire (Sleep Recovery Scale, SRS_HRI_ [32]) and to assess their current feeling of well-being (Basler well-being score [31]). At the end of each three-week experimental sequence, the habitual sleep quality was assessed with the Pittsburgh Sleep Quality Index (PSQI [30]).

### 2.7. Statistical Evaluation

General linear models (GLMs) were used to perform a per-protocol analysis with repeated measures ANOVA (Table 1, Table 2 and Table 3). The within-subject factor was the “treatment (bed)” (stone pine vs. chipboard) at different times of the day (sleep vs. wake; sleep epochs) or scales of the questionnaires, which were aggregated for six repeated measurements for each subject and treatment. The calculation of the ‘sleep’ and ‘wake’ periods used was performed on the basis of the activity protocols of the subjects, which were visually checked for their plausibility, whereby transitions between wake and sleep (i.e., the first and last 30 min of each activity period) were not taken into account. In addition to the *p*-values, the effect size, partial Eta^2^ (η^2^) was calculated. An η^2^ of 0.01–0.06 corresponds to a small effect. Occurrences of 0.06–0.14 correspond to a medium effect, and values > 0.14, to a large effect [45]. For the purposes of statistical analysis, missing or invalid values of HRV parameters were replaced by interpolated means of adjacent data points in 4.4% (24 h-HRV) and 9.7% (sleep-HRV) of all cases. Calculations were performed with IBM^®^ SPSS^®^ Statistics (Version 22) (IBM Corporation, Armonk, NY, USA).

## 3. Results

### 3.1. Heart Rate and Vagal Activity

**Figure 3:**
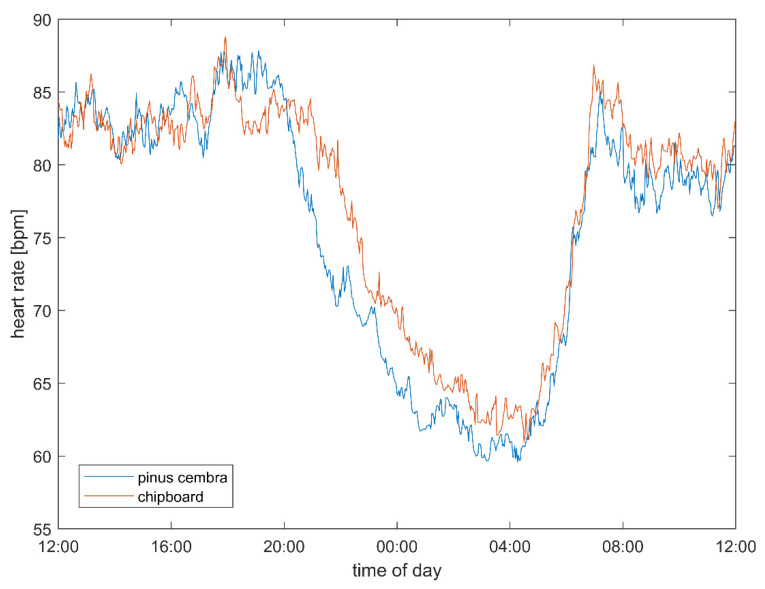
Heart rate over 24 h when the subjects slept in the stone pine wood beds (blue) or in or in the chipboard beds (red). Each curve represents the average of nearly 90 days of measurements for 15 subjects. The strongest difference in the heart rate was found during the first phase of sleep.

The subjects’ heart rate was found to be reduced when sleeping as compared to in the daytime, and this reduction was larger if the same subject slept in a bed made out of stone pine wood than if they slept in one made out of chipboard (Figure 3) or in their own bed (data not shown). The greatest changes and differences between the two bed types were found in the first two hours of sleep.

**Figure 4:**
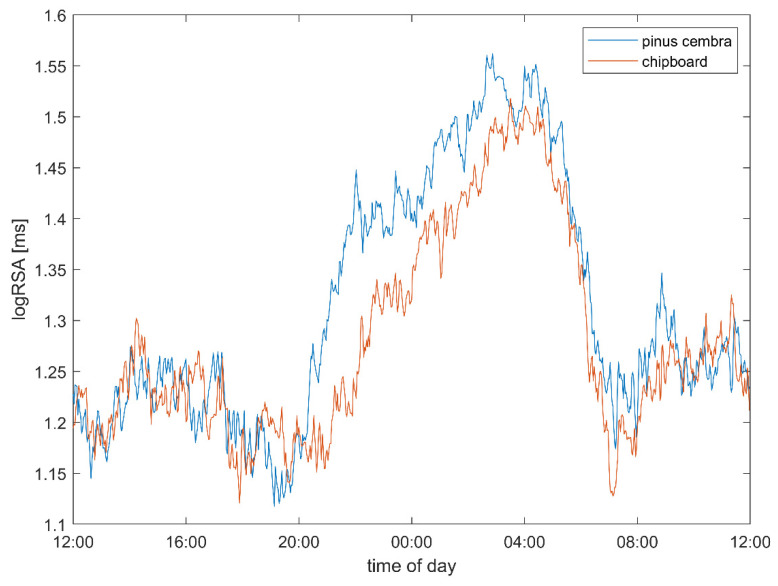
Vagal activity as computed from the respiratory component of HRV (logRSA), when the subjects slept in the stone pine wood beds (blue) and in the chipboard beds (red). Each curve represents the average of nearly 90 nights of measurements for 15 subjects. Like in the heart rate, the strongest difference in vagal activity was found during the first hours of sleep.

Vagal activity (logRSA) was found to be increased when sleeping as compared to in the daytime, and this increase was larger if the same subject slept in a bed made out of stone pine wood than if they slept in one made out of chipboard (Figure 4) or in their own bed (data not shown). As with heart rate changes, the largest differences were found during the first hours of sleep.

Table 1 shows an overview of the heart rates, CRIs (logRSA, pulse–respiration quotient) and other autonomic parameters for the two bed types for waking and sleeping periods, as well as over 24 h.

The heart rate (stone pine vs. chipboard: 74.09 vs. 76.61 bpm, *p* = 0.008; Table 1), vagal tone (1.31 vs. 1.27 log(ms), trend: *p* = 0.057) and pulse–respiration quotient (5.03 vs. 5.29 beats per respiratory cycle, *p* = 0.037) show multivariate significant differences between the two bed conditions, which are strongest during sleep (Table 2). These differences can be also observed over the 24 h and wake periods as well. In addition, a trend towards higher total heart rate variability (SDNN: 73.50 vs. 70.23 ms, ns.; total variability power 8.14 vs. 8.07 ln(ms^2^), ns.) in the stone pine bed condition can be observed. The autonomic quotient (LF/HF: 0.89 vs. 0.93, ns.) and low frequency power (6.77 vs. 6.72 ln(ms^2^), ns.) are both lower but not significantly lower between the experimental conditions, indicating a shift from sympathetic towards parasympathetic predominance. The pulse–respiration quotient is significantly lower and closer to 4:1 in the stone pine bed.

During the core sleeping period (Table 2), i.e., the first three hours of sleep in each subject, the heart rate (stone pine vs. chipboard: 62.73 vs. 65.69 bpm, *p* = 0.012), vagal tone (1.49 vs. 1.42 log(ms), *p* = 0.042) and pulse–respiration quotient (4.08 vs. 4.25 beats per respiratory cycle, *p* = 0.027) show stronger and significant differences between the two bed conditions. During this core sleep, an even stronger trend towards higher total heart rate variability can be observed (SDNN: 71.14 vs. 64.71 ms, ns.; total variability power 8.62 vs. 8.44 ln(ms^2^), ns.) in the stone pine bed. The autonomic quotient (LF/HF: 0.17 vs. 0.23, ns.) and low frequency power (7.22 vs. 7.06 ln(ms^2^), ns.) are both lower but not significantly lower between the experimental conditions, indicating a shift towards parasympathetic predominance. The pulse–respiration quotient is significantly lower and closer to 4:1 in the stone pine bed.

### 3.2. Psychometric Results

No differences were observed in the frequency of stress and recovery-related activities and states (RESTQ) between the sleeping periods in the two bed types (*p* > 0.50), indicating that the subjects experienced similar levels of stress during the periods under both experimental conditions. Other than in the physiological results, no significant difference was detected in subjective sleep quality and with the sleep recovery scale, but the subjects experienced significantly better feelings of subjective well-being as measured with the BBS (Basler–Befindlichkeits-Skala) overall scale (0.16 vs. −0.18 units, *p* = 0.020) and the interpsychic stability subscale (0.16 vs. −0.25 units, *p* = 0.023) in the morning after sleeping in the stone pine bed than after sleeping in the chipboard bed. An almost significant trend towards higher social extraversion as measured by BBS was also detected after subjects slept in the stone pine bed (0.19 vs. −0.19 units, *p* = 0.051).

### 3.3. Pulse–Respiration Quotient

**Figure 5:**
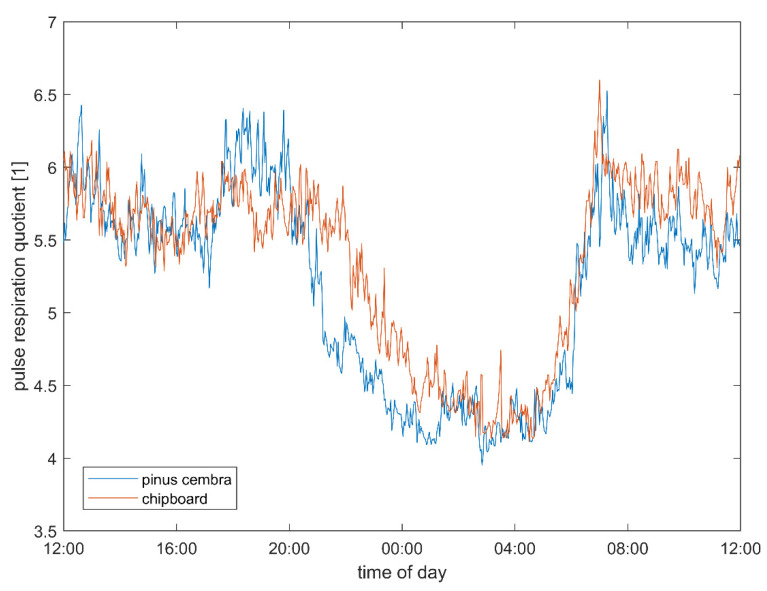
Pulse–respiration quotient (Qpr) as computed from the Holter ECG recordings, when the subjects slept in the solid wood beds (blue) and in the chipboard beds (red). Each curve represents the average of 90 nights of measurements for 15 subjects. The Qpr is higher during the day and approaches a relation of four heartbeats per respiratory cycle during the night. If the subjects slept in the stone pine bed, 4:1 was reached about two hours earlier than if they slept in the control bed. As seen for the heart rate and the vagal activity, the strongest difference in the pulse–respiration quotient was found during the first phase of sleep. The effect of lower Qpr also persists in the morning after the waking.

The pulse–respiration quotient (Qpr) was closer to 4:1 and lower during sleep than in the daytime, and these effects were larger if the same subject slept in a bed made from stone pine wood than if they slept in either the chipboard bed (Figure 5). This reduction in the Qpr after sleeping in the stone pine bed also persisted in the morning until the following noon (see also ‘autonomic quotient interaction’).

### 3.4. Autonomic Quotient Interaction

**Figure 6:**
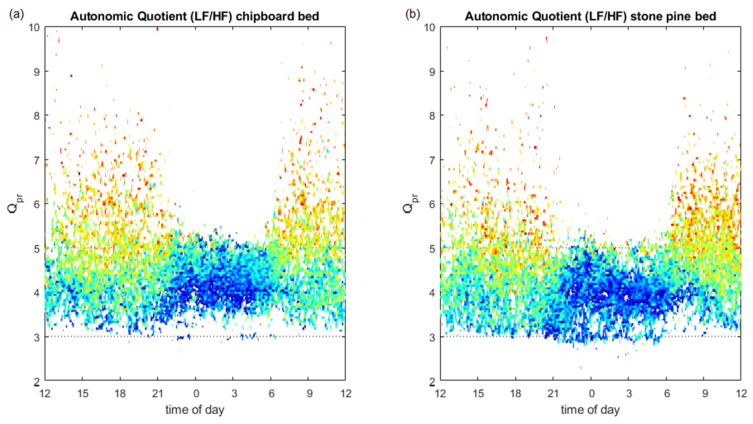
Average of autonomic quotient (LF/HF, colour coded: red = sympathetic predominance, blue = vagal predominance) over the time of day (abscissa) and pulse–respiration quotient (Qpr, ordinate), when the subjects slept in the stone pine wood beds (**b**) and in the chipboard beds (**a**). The Qpr is higher during the day and approaches a relation of four heartbeats per respiratory cycle during the night. A whole number Qpr of 4:1 and 3:1 is coincident with an especially low autonomic quotient (coded in blue), indicating strong vagal predominance. The 4:1 period of Qpr with stronger vagal predominance lasts two hours longer during and after sleeping in the stone pine bed (**b**), indicating a longer period of phase coupling for cardiorespiratory interactions (CRIs) in this type of bed.

The autonomic quotient (LF/HF) changed during the diurnal cycle, from higher and more distributed values during the day to lower and less distributed during the night (Figure 6). When a pulse–respiration quotient around whole number ratios (especially 4:1) is present, indicating transient phase coupling between pulse and respiration, the autonomic quotient becomes especially low, indicating vagal predominance (dark blue areas). The 4:1 period of Qpr with stronger vagal predominance lasts 2 h longer in the stone pine bed (Figure 6b).

### 3.5. Sleep Architecture

**Figure 7:**
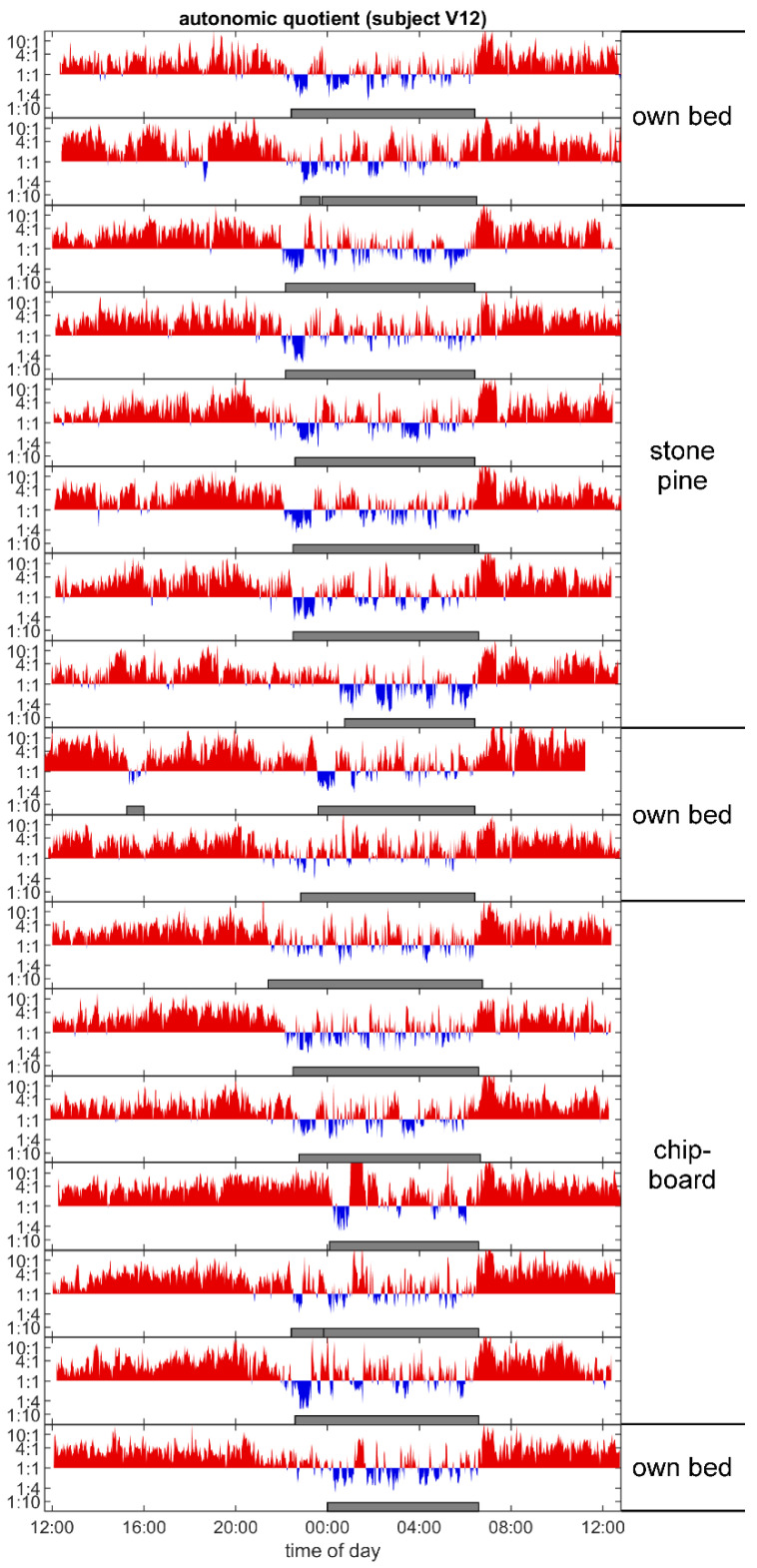
Example of the circadian and ultradian changes in the autonomic quotient over all measured nights for one subject (Number 12) during the study. After two measurements were made in their own bed, six measurements over three weeks were made in the stone pine bed. Two measurements were then made over the six-week washout period in their own bed, then another six measurements over three weeks were performed in the control bed (chipboard). The last measurement was made in the subject’s own bed again. Sympathetic predominance is shown in red, and vagal predominance, in blue. Especially during the period in the stone pine bed, the first non-REM (core sleep) phases are more rhythmic and more vagal (blue) than under the control (chipboard) bed conditions.

Sleep architecture, as seen in the individual time course of the autonomic quotient over the different measured nights, was found to be more qualitatively, regular during the periods of sleeping in the *Stone pine* beds than when sleeping in the chipboard beds (example, see Figure 7). In the former cases, the basal rest and activity cycles (duration 1.5 to 2 h) seem to be more dominant and regular in subjects who slept in the stone pine beds. A stronger oscillation between vagal and sympathetic predominance with stronger vagal phases (blue) was also present in the subjects who slept in the *Pinus cembra* beds.

**Figure 8:**
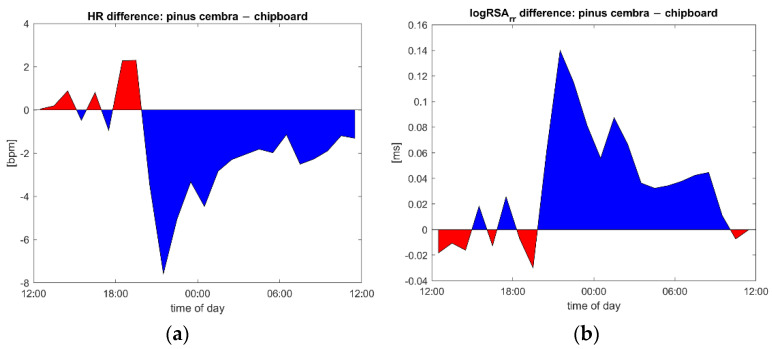
(**a**) Time course of heart rate differences between sleeping in the stone pine (*Pinus cembra*) bed and sleep and the control condition (chipboard bed). Blue indicates lower heart rate in the stone pine bed and red, the lower heart rate in control condition. (**b**) Differences in the vagal tone (logRSA). Here, blue indicates a higher vagal tone in the stone pine bed and red, under the control condition.

Both the decrease in the heart rate (Figure 3) and the increase in the vagal activity (Figure 4) were significantly stronger when the subjects slept in the stone pine bed than when they slept in the chipboard bed. This indicates an improvement in CRIs, and especially in respiratory sinus arrythmia, which positively correlates with the use of stone pine wood in the bed. To illustrate the timing of the observed effects, we plotted the difference between the stone pine and control conditions, using the values in the control bed as a zero reference (Figure 8). In fact, the difference in the heart rate (Figure 8a) and vagal tone (Figure 8b) became obvious only after 8 p.m., when the subjects entered the bedroom, and lasted until noon the next day.

## 4. Discussion

The materials used indoors interact with the environment in which the occupants live. Human satisfaction and interactions with the built environment on cognitive functions, well-being and health have become increasingly relevant [46,47,48]. Efforts have been made to predict human well-being and physiological responses to better understand how the physical environment directly impacts human well-being, health and productivity [49]. Recent studies have shown that the use of natural materials in indoor built environments such as wood can improve human well-being.

### 4.1. Experimental Design

Findings from recreation research show that sustainable adjustments in the autonomic system evolve over a period of several weeks [50]. Therefore, the intervention period was selected to last for three weeks. Given this premise, a sleep study is practically feasible only in a home setting; therefore, this setting was chosen.

### 4.2. Heart Rate and Autonomic Parameters

In this study, it was possible to use state-of-the-art, high-precision ECG devices to record and detect R peaks as well as to compute the heart rate variability parameters. The observed reduction in the heart rate during sleep indicates a reduction in metabolic activity and a reduction in the heart’s workload during the night. This mirrors the increase in vagal activity, indicated by an increased respiratory component of the heart rate variability (logRSA). The heart rate is actually decreased by vagal activity, so the observed reduction in the heart rate is believed to result from an increase in vagal activity. Both effects were the most strongly pronounced during the first hours of sleep in the study beds, indicating a mutual interdependence.

These results (e.g., Figure 8) indicate that the use of the stone pine wood may positively contribute to the vagostimulant, and heart-rate-reducing effects observed in this study. Other studies have found that vagal activity can be increased by volatiles exuded from pinewood due to olfactory stimulation [12,51,52,53,54] as well as by physical contact with the wood [13,14].

### 4.3. Possible Health Consequences

The immune system issues a “license to kill” when inflammatory processes are triggered in reaction to infections and dangerous cell mutations that might cause cancer [55]. Although this response is a live-saving measure under normal conditions, inflammation can become chronic even in the absence of infectious agents for reasons that are still unknown. This state is known as “silent inflammation” and is present in the onset of many chronic diseases, including atherosclerosis [56], depression and Parkinson’s disease [57] and even cancer [58]. This trend is so worrying that *Nature* first issued a Special Issue in 2002 to illustrate these connections [59], which promoted by many other medical studies on the topic. Vagal activity has been also related to inflammatory control, and studies have been carried out to elucidate interaction pathways between sensory and motor vagal neurons and immune cells [26]. In a control loop termed the “vagal inflammatory reflex” [26], macrophages in the inflamed tissue produce inflammation signals such as TNF-alpha and interleukin 1 [60] which attract other monocytes from nearby blood vessels. Vagal afferents carry receptors for these signals and communicate with certain stem brain areas, transmitting information about the location and strength of the inflammation. Upon processing this information, vagal efferents respond by locally releasing acetylcholine within the inflamed tissue. Nicotinergic acetylcholine receptors have been identified on the macrophage surfaces, which downregulate their cytokine production in response to the cholinergic stimulation [61], thereby reducing the attraction of additional inflammatory immune cells. This inflammatory reflex loop prevents the overstimulation of the immune system, enabling the brain to locally control the immune activity. It also represents the “first line” of inflammation control [26]. This information can be applied to better understand the observation illustrated in Figure 6, which shows that the vagal stimulation effect is especially strong at night, when the immune system is the most strongly active and vagal inflammation control is most strongly needed. At this time, the pulse–respiration quotient is at a whole number ratio, indicating the establishment of a stable phase relationship between the cardiac and respiratory cycles. The findings of this study regarding the positive effects of sleeping in stone pine beds are supported by those of other studies, which support the positive and anti-inflammatory health effects of olfactory contact with volatile pine terpenes with [16,62,63,64,65]. These effects may also be due to their vagally stimulating action: Pine terpenes have been shown to have a variety of effects, from simple stress relief to reductions in cancer rates. The U.S. Department of Health itself has filed a patent with a claim pine terpenes can be used to treat cancer [66].

Such studies, which highlight the importance of exposure to natural phytochemicals in nature, also underline the importance of the preservation of urban green space, and especially trees in these spaces, to support human health. This is a particularly exciting emerging field of public health [67,68]. Access to trees in public areas has drawn large amounts of attention in the recent past, when a study was carried out to investigate the effects of this access on more than 46,000 subjects after adjusting for age, sex, income, economic status, couple status and educational level. The findings indicated that subjects who had access to areas with >30% tree canopy coverage, as compared with subjects who only had access to areas with up to 9% tree canopy coverage, had a 31% lower incidence of psychological distress. This effect, however, was not found with regard to the proximity to lawns or shrubs [69]. Tree cover on campus that accounted for 13% of the variance could be used to predict better mean student performance in another study [70]. Further, an increase in the percentage of tree canopy coverage in a census block group was associated with a lower risk of experiencing short sleep periods (<6 h) during weekdays (OR 0.76 [0.58–0.98]) and, hence, with better overall sleep quality [71].

In this study, although we have not investigated the effect of living trees on the subjects, the volatile phytochemicals present in trees as well as in the solid wood from these trees might at least contribute to the described health effects. One very interesting aspect was added to the importance of green space, when a Lancet study of 360,000 British people showed that populations exposed to the greenest environments also have the lowest levels of health inequality with respect to income deprivation [72]. The same study showed that especially the number of cardiovascular diseases suffered by members of lower income classes were reduced by green space access. As vagal activity obviously protects the heart from arterial fibrillation, these findings support the observed vagal effects of access to green space and maybe to indoor built environments that include natural materials [23].

To investigate the possible amount of improvement that could be attained by reducing the heart rate by 2.52 beats per min (average 24-h-HR = 76.61 vs. 74.09 in our study), we searched for studies that associated the heart rate with specific mortality rates. In a study with nearly 240,000 patients, Archangelidi et al. [73] found that men with a resting heart rate of 70–79 bpm (29.1% of all men) had a higher risk of heart failure (hazard ratio (HR) 1.65, 95% confidence interval (CI) 1.26–2.16), unheralded coronary death (HR 1.65, 95% CI 1.13–2.41), total cardiovascular events (HR 1.22, 95% CI 1.15–1.28) and even all-cause mortality (HR 1.39, 95% CI 1.22–1.58) than men with a resting heart rate of less than 60 bpm. This connection between a low heart rate and lower mortality has been confirmed by several other studies [74,75,76]. In a linear model, a reduction of the heart rate by 2.52 beats per min actually would result in a reduction of mortality by approximately 10%. As we cannot be sure that the observed effect on heart rate will persist over a period of several years, and more support is needed for a purely linear correlation (although some support has been provided by the results of the 25-year Paris Prospective Study I with 6101 asymptomatic men aged 42 to 53 years [75]), these calculations might be premature. However, these findings show the health potential of reducing the heart rate by promoting the use of an indoor built environment with natural materials, such as the stone pine beds used in this study.

### 4.4. Pulse–Respiration Quotient

The pulse–respiration quotient (Qpr), the number of heartbeats per respiratory cycle, has been identified as a powerful but rarely used parameter in the field of physiology [18,24,43]. In some diseases, such as myocardial infarction and hyperthyroidism [24], an increased Qpr and a reduced circadian variation have been documented. In normal subjects, the Qpr is higher when they are in the upright, rather than in the supine, position [24], which explains the circadian time course illustrated in Figure 5. This figure also shows that sleeping in a stone pine bed obviously leads to an earlier fall in Qpr as compared to sleeping in the chipboard beds. This lowering effect of this wood persists until the following noon, resulting in significant all-over lowering effect on Qpr (Table 1).

### 4.5. Relation of Autonomic Quotient to Daytime and the Pulse–Respiration Quotient

The change in the autonomic quotient (LF/HF) that naturally occurs during the circadian cycle, ranging from higher and more evenly distributed values during the day to lower and less evenly distributed values during the night (Figure 6), could also be documented in this study. Whole number ratios (especially 4:1) arise when phase coupling between the cardiac cycle and the respiratory phase occurs, as the cardiac phase may trigger the onset of inspiration [18]. If this is repeatedly the case, the heart rate becomes a whole-number multiple of the inspiratory cycle. Studies have shown that this phase coupling is especially strong during periods of relaxation and sleep. Under stress and in psychiatric diseases like depression, phase coupling might even disappear [77,78]. Obviously, the autonomic quotient drops especially low when this phase coupling arises, indicating a strong vagal predominance, shown as blue areas that surround the 4:1 Qpr in Figure 6. This ‘blue area’ lasted about two hours longer in subjects who slept in the stone pine bed (Figure 6b) than in subjects who slept in the chipboard bed (Figure 6a).

### 4.6. Autonomic Sleep Architecture

Over a period of 24 h, the autonomic quotient (LF/HF) does not only change according to a circadian cycle, but also shows a ultradian pattern (period length 1.5–2 h) [79], which corresponds to the BRAC (basic rest and activity cycle) as documented in sleep research [80]. This pattern also can be observed in persons experiencing healthy sleep and is an indicator of a sleep architecture that characterizes good sleep [81]. As such, it can be used as a qualitative measure of (autonomic) sleep quality. When we plotted the autonomic quotient time series of each subject and categorized the data by the different type of bed, we realized that subjects who slept in the stone pine beds usually experienced more structured sleep and showed a more pronounced sleep architecture than those who slept in the chipboard beds. An example of this observation is shown in Figure 7, where a stronger vagal predominance during all of the first sleep phases in the stone pine bed is also present. Although we did not test this difference statistically, it could be considered as an indication of qualitative improvement in the sleep architecture of those subjects who slept in the stone pine bed.

### 4.7. Limitations

In addition to the small sample size, one limitation of this study might be the lack of knowledge about the volatile organic compound (VOCs) compositions and concentrations in the bedrooms of the study participants, as the most likely factor influencing the observed effects. As this study was intended to investigate the overall effect of different indoor settings under real-life conditions, only the bed material was changed in the everyday sleeping conditions. Therefore, a within-subject design with numerous repeated (aggregated) measurements was chosen to reduce the possible moderating factors of the individual sleep environment, such as specific biogenic VOCs concentrations. In this setting, these are not as crucial as would have been the case if a comparison had been carried out between different subjects. Future studies may focus on the exact concentrations of any VOCs and singular components when exposure occurs. Besides the exact concentrations of VOCs, the duration of exposure, setting, individual baseline status, health-characteristics and lifestyle habits may influence the impact of VOCs [11]. The potentially adverse effects of volatile biogenic substances should also be taken into account [10]. Adverse effects were not observed in our study and are not to be expected in contact with untreated natural substances, if they occur in a similar composition and concentration as in natural environments.

Integration of polysomnography (PSG) as the gold standard of sleep medicine (central nervous system modulation) would have been interesting, although in our opinion PSG indices are not sensitive enough to enable the detection of the effects of environmental conditions or of VOCs. Autonomic nervous system modulation such as CRIs and vagal activity appear better suited to determining the environmental effects on the human organism, which can be achieved by analyzing the heart rate variability (HRV) and which mirrors autonomic activities generated in lower stem brain centers [82]. In addition, PSG measurements would have been beyond the scope of the study, as they are technically complex and costly and take place mainly under laboratory conditions. They also are known to disturb the sleep (first night effect) due to the presence of several electrodes on the scalp, which was not observed in our three-chest wall ECG electrode design.

## 5. Conclusions

In this study, we show that CRIs are a sensitive measure that can be used to describe the effects of the indoor built environment on physiological responses of autonomic nervous activity during sleep. The study subjects’ vagal activity increased, especially when sleeping, whereas their heart rate and pulse–respiration quotient decreased, when they slept in a bed made from stone pine (*Pinus cembra*) wood. CRIs increased and extended longer into the morning, suggesting that better tuning between the two organismic oscillators of heartbeat and respiration occurred when subjects slept in a stone pine bed. These findings offer new insights into the topic of why a better built living environment should be created.

In the view of recent findings on the importance of low heart rate for life expectancies and on the importance of high vagal activity with regard to inflammatory control and the resolution of silent inflammation, the study results tentatively explain why stone pine wood has been used traditionally and for many centuries as a preferred material for building beds in areas where the tree is indigenous.

## Figures and Tables

**Table 1:** Heart rate, cardiorespiratory interaction (CRIs) and autonomic measures—24 h, awake and asleep in different types of beds.

*Heart Rate Variability (HRV)* (*n = 15*) *		P. Cembra (24 h; 6×)			Chipboard (24 h; 6×)			Sleep (Mean; 6×)		Wake (Mean; 6×)		Bed Effect (2 × 2 ANOVA) (P. Cembra vs. Chipboard)		
	Unit	Mean		SD	Mean		SD	P. Cembra	Chip-Board	P. Cembra	Chip-Board	F	*P*	Part. Eta^2^
**Heart rate (HR)**	bpm	74.09	±	7.42	76.61	±	7.39	60.91	63.76	79.97	82.18	9.56	0.008 **	0.41
**Standard deviation of RR (SDNN)**	ms	73.50	±	22.04	70.23	±	20.03	77.12	71.91	71.81	69.01	3.01	0.105	0.18
**Vagal tone (logRSA_rr_)**	log(ms)	1.31	±	0.18	1.27	±	0.16	1.50	1.45	1.23	1.20	4.32	0.057 *	0.24
**Total variability power (lnTOT_rr_)**	ln(ms^2^)	8.14	±	0.58	8.07	±	0.53	8.14	8.01	8.15	8.08	2.65	0.126	0.16
**Low frequency power (lnLF_rr_)**	ln(ms^2^)	6.77	±	0.68	6.72	±	0.62	6.72	6.59	6.81	6.77	1.75	0.207	0.11
**High frequency power (lnHF_rr_)**	ms^2^	5.87	±	0.81	5.78	±	0.75	6.51	6.34	5.60	5.54	2.10	0.169	0.13
**Very low frequency power (lnVLF_rr_)**	ms^2^	7.39	±	0.51	7.30	±	0.47	7.14	7.01	7.50	7.41	3.18	0.096	0.19
**Ratio LF/HF**	[ ]	0.89	±	0.47	0.93	±	0.47	0.21	0.25	1.21	1.23	0.46	0.509	0.03
**Pulse-respiration quotient (Qpr)**	bpc	5.03	±	0.89	5.29	±	0.91	4.09	4.27	5.45	5.72	5.30	0.037 *	0.28
**Respiratory rate (ATMF_rsa_)**	fpm	15.60	±	2.05	15.42	±	2.01	15.10	15.13	15.84	15.56	0.45	0.514	0.03

*. Missing = 4.44% …. out of overall 15 × 6 × 2 = 180 × 24 h HRV-measurements, ** multivariate significant difference.

**Table 2:** HRV—core sleep in different bed materials.

*Heart Rate Variability (HRV)* *(n = 15; Sleep Measurements = 180 with Each 6 Epochs)* *		P. Cembra (Sleep Mean 30 min Epochs)						P. Cembra (Core Sleep; 3 h)		Chipboard (Sleep Mean 30 min Epochs)						Chipboard (Core Sleep; 3 h)		Mean Difference (P. Cembra - Chipboard)		Bed Effect (2[×6] ANOVA) (P. Cembra vs. Chipboard)		
	unit	1	2	3	4	5	6	Mean	SD	1	2	3	4	5	6	Mean	SD	Mean	SE	F	*P*	Part. Eta^2^
**Heart rate (HR)**	bpm	64.04	62.74	63.30	63.04	61.77	61.47	62.73	8.47	67.01	66.00	67.11	65.52	64.43	64.07	65.69	7.28	−2.96	1.03	8.33	0.012*	0.37
**Standard deviation of RR (SDNN)**	ms	74.87	68.25	71.51	68.12	71.71	72.38	71.14	25.83	62.57	57.86	62.82	60.75	67.03	77.22	64.71	26.20	6.43	3.67	3.07	0.102	0.18
**Vagal tone (logRSA_rr_)**	log(ms)	1.51	1.53	1.48	1.47	1.46	1.46	1.49	0.24	1.43	1.43	1.37	1.39	1.45	1.43	1.42	0.21	0.07	0.03	5.03	0.042*	0.26
**Total variability power (lnTOT_rr_)**	ln(ms^2^)	8.73	8.53	8.59	8.59	8.65	8.64	8.62	0.75	8.40	8.18	8.44	8.34	8.50	8.77	8.44	0.70	0.18	0.10	3.18	0.096	0.19
**Low frequency power (lnLF_rr_)**	ln(ms^2^)	7.32	7.13	7.18	7.20	7.26	7.24	7.22	0.89	7.01	6.77	7.14	7.00	7.13	7.33	7.06	0.84	0.16	0.11	2.19	0.161	0.14
**High frequency power (lnHF_rr_)**	ms^2^	7.24	7.18	7.02	6.97	6.99	6.95	7.06	1.01	6.93	6.81	6.66	6.73	6.91	6.94	6.83	0.88	0.23	0.13	3.14	0.098	0.18
**Very low frequency power (lnVLF_rr_)**	ms^2^	7.62	7.30	7.49	7.54	7.59	7.66	7.53	0.68	7.23	6.93	7.45	7.26	7.40	7.85	7.35	0.67	0.18	0.09	3.80	0.072	0.21
**Ratio LF/HF**	[ ]	0.08	−0.05	0.17	0.23	0.27	0.30	0.17	0.75	0.07	−0.04	0.48	0.27	0.22	0.39	0.23	0.73	−0.07	0.06	1.17	0.298	0.08
**Pulse-respiration quotient (Qpr)**	bpc	4.16	4.04	4.08	4.06	4.09	4.03	4.08	0.50	4.32	4.21	4.33	4.25	4.18	4.21	4.25	0.40	−0.17	0.07	6.13	0.027*	0.30
**Respiratory rate (ATMF_rsa_)**	fpm	15.76	15.83	15.77	15.69	15.26	15.48	15.63	1.86	15.78	15.93	15.77	15.57	15.69	15.40	15.69	1.70	−0.06	0.14	0.16	0.694	0.01

*. Missing = 9.67% … out of overall 1080 sleep epochs.

**Table 3:** Questionnaire responses—psychometric characteristics (traits and states) in different beds.

*Scale* *(n = 15; Surveys = [30 to] 180)* *	[Unit]; Frequency	P. Cembra (3 Weeks)			Chipboard (3 Weeks)			Mean Difference P. Cembra - Chipboard		Bed Effect (2 Groups Design) (P. Cembra vs. Chipboard)		
		Mean		SD	Mean		SD	Mean	SE	F	*P*	Part. Eta^2^
**PSQI (n = 12)**	[ ]; 2×	4.25	±	2.34	3.83	±	2.32	0.42	0.63	0.43	0.524	0.04
**Sleep Recovery Scale (SRS_HRI_, main factor; n = 14)**	[z], 12× (6 times each)	0.06	±	0.54	−0.07	±	0.63	0.13	0.23	0.33	0.575	0.03
sleep characteristics (SRS, Factor [F]1)		0.05	±	0.54	0.00	±	0.63	0.04	0.17	0.07	0.795	0.01
sleep quality (F2)		0.01	±	0.62	−0.04	±	0.64	0.04	0.22	0.04	0.850	0.00
sleep time (F3)		0.08	±	0.69	−0.11	±	0.61	0.19	0.24	0.63	0.440	0.05
**Well-being/mood (BBS, total; n = 14)**	[z], 12× (6 times each)	0.16	±	0.74	−0.18	±	0.78	0.34	0.13	7.01	0.020 *	0.35
intrapsychic stability		0.16	±	0.51	−0.25	±	0.89	0.40	0.16	6.64	0.023 *	0.34
vitality		0.12	±	0.70	−0.10	±	0.78	0.23	0.16	1.96	0.185	0.13
social extraversion		0.19	±	0.86	−0.19	±	0.65	0.38	0.18	4.63	0.051 *	0.26
vigility		0.06	±	0.78	−0.09	±	0.80	0.15	0.09	2.70	0.125	0.17
**Recovery-Stress Q. (RSTQ-Basic-24, multivariat, n = 15)**	[z], 12× (6 times each)									0.20	0.826	0.03
stress scales		−0.04	±	0.84	−0.04	±	0.83	0.01	0.08	0.01	0.946	0.00
recovery scales		−0.02	±	0.90	0.08	±	0.69	−0.10	0.16	0.39	0.540	0.03

*. Missing: 13.33% PSQI (out of 30 measures); 17.78% SRS, 23.33% BBS, 7.22% RSTQ …. out of overall 180 surveys. Scales (questionnaires): PSQI [30], SRS_HRI_ [32], BBS [31] & RSTQ-Basic-24 [33,34].

## Data Availability

The data presented in this study are available on request from the corresponding authors. The data are not publicly available due to ethical and legal restrictions. The data contains potentially identifying and sensitive information about individuals.
